# Molecular identification and targeting of colorectal cancer stem cells

**DOI:** 10.18632/oncotarget.173

**Published:** 2010-10-09

**Authors:** Kristel Kemper, Catarina Grandela, Jan Paul Medema

**Affiliations:** LEXOR (Lab for Experimental Oncology and Radiobiology), Center for Experimental Molecular Medicine, Academic Medical Center, Amsterdam, the Netherlands

**Keywords:** colon cancer stem cells, markers, targeting

## Abstract

Tumor initiating or cancer stem cells (CSCs) are suggested to be responsible for tumor initiation and growth. Moreover, therapy resistance and minimal residual disease are thought to result from selective resistance of CSCs. Isolation of CSCs from colon carcinomas can be accomplished by selection of a subpopulation of tumor cells based on expression of one or multiple cell surface markers associated with cancer stemness, like CD133, CD44, CD24, CD29, CD166 and Lgr5. Identification of colon CSCs will lead to a better rational for new therapies that aim to target this fraction specifically. In this review, we analyze known markers used for selection of colon CSCs and their potential function in CSC biology. Moreover, we discuss potential targeting strategies for eradicating CSCs specifically in order to develop more effective therapeutic strategies as well as to address more fundamental questions like the actual role of CSCs in tumor growth.

## INTRODUCTION

Tumors have long been viewed as a population in which all cells have the equal propensity to form new tumors, the so called conventional stochastic model. New insight was obtained when a small subset was discovered in acute myeloid leukemia (AML) [1,2] that retained the ability to serially transplant. Since then, a new hierarchical model was born, claiming that tumors consist of a small fraction of so called tumor initiating cells, also referred to as cancer stem cells (CSCs) that are capable of initiating and maintaining tumor growth and a large fraction of more differentiated cells, which are incapable of maintaining tumor growth. After discovering these CSCs in hematological malignancies, their presence was also found in several solid tumors, like breast [3-5], lung [6], ovarian [7], liver [8], prostate [9], pancreas [10], skin [11], brain [12,13] and colon cancer [14-17], which will be the focus of this review. To prove the presence of CSCs within a tumor, several main criteria have been established by the scientific community: firstly, CSCs, identified by a specific cell surface marker, should be able to serially transplant the tumor in vivo; secondly, the tumors that grow out from the CSCs should resemble the original malignancy; thirdly, CSCs should be able to differentiate in marker-negative cells. To determine the CSC-frequency, in vivo limiting dilution assays (LDA) are performed, which means that several dilutions and multiple replicates of marker-positive or marker-negative cancer cells are injected subcutaneously in (immunodeficient) mice. Cell surface markers that have been used for identification of colon CSCs are CD44 [18-20], CD133 [14-17], CD166 [20], whereas expression of CD24 [17], CD29 [17] and Lgr5 [21] has also been found in colon CSCs. More functional markers like Wnt activity [22] and ALDH1 activity [23] have been exploited as well. Through identification of CSCs in colon cancer, more can be learned about the role of these cells in initiating and driving tumor growth. In this review, we examine the markers used for colon CSC identification and their possible function, as well as methods to target these CSCs specifically, both at therapeutic as wells as at a more fundamental level.

## MARKERS USED TO IDENTIFY COLON CSC

The five-transmembrane glycoprotein CD133 is one of the first colon CSCs markers identified and its use as a CSC marker has been controversial since then. Selecting colon cancer cells based on positivity for AC133, an epitope on the CD133 protein, identifies the tumorigenic and clonogenic population [14-17,24]. On the other hand, CD133 expression was found throughout the normal gastro-intestinal tract and therefore does not seem to be restricted to the stem cell compartment [25,26]. In addition, CD133^+^ as well as CD133^−^ metastatic colon cancer cells were shown to be able to form new tumors, indicating that usage of CD133 as a CSC marker is questionable [26]. In this light, it is important to note two things. First, CD133 expression can be regulated by hypermethylating the CpG island in the CD133 promoter region, an event that frequently occurs in higher grade tumors and results in CD133 downregulation independent from its potential role in CSCs [27]. Secondly, the CD133 mRNA or surface expression is not changed during differentiation [24,28]. Instead the AC133 epitope is masked and inaccessible for the antibody in differentiated colon cancer cells, likely due to a difference in the glycosylation status and folding of the protein [24]. In agreement, the epitope can be re-exposed by certain treatments, potentially explaining different outcomes in immunohistochemical stainings [24,26]. Although CD133 can be used as a CSC marker, it therefore should be done with caution.

The transmembrane glycoprotein CD44 has been used as a marker to isolate CSCs from multiple solid tumors, such as breast [3], head and neck [29], pancreas [30] and also colon cancer [31].Colon cancer cells sorted for CD44^+^ displayed high tumorigenicity, especially in combination with CD133-positivity, whereas CD44^−^ cells could not form new tumors in immunodeficient mice [19,20]. CD44 can also be used in combination with mesenchymal stem cell marker CD166 (also referred to as ALCAM). CD44^+^CD166^+^ colon cancer cells display a higher ability to form tumors in immunodeficient mice as compared to CD44^+^CD166^−^, CD44^−^CD166^−^ or CD44^−^CD166^−^ cell populations [20], making this an useful combination for the identification of colon CSCs.

In normal colon, integrin subunit β1 (CD29) expression was observed in the lower parts of the crypt and thereby seems to mark a more stem/progenitor cell type [32]. Moreover, the combination of CD24^high^CD29^high^ was suggested to identify the tumor initiation fraction in mouse colon carcinomas (R.Fodde, personal communication, [17]). Indeed, human colon CSCs were found to express high levels of CD24 and CD29 [17], suggesting that these markers can also identify human colon CSCs, although further evidence is necessary.

Not only cell surface markers, but also activity of certain pathways or enzymes can mark stemness. For instance, normal colon stem cells can be identified by the activity of aldehyde dehydrogenase 1 (ALDH1), a detoxifying enzyme that oxidizes intracellular aldehydes, therefore involved in the resistance to alkylating enzymes. ALDH1 activity can be measured by a method using dansyl aminoacetaldehyde and staining for ALDH1 revealed a few positive cells in the crypt [23]. In addition, when colon cancer cells were selected for ALDH1 activity using Aldeflour, ALDH1^+^ cells were able to initiate new tumors whereas ALDH1^−^ cells were not [23], indicating that ALDH1 activity can be used as a colon CSC marker.

Besides the ALDH1 activity, recent evidence indicates that Wnt signaling activity can serve as a functional designation of colon CSCs [22]. Activating mutations in the Wnt pathway are pivotal in the initiation of the majority of colon cancers. However, recent evidence indicates that a clear heterogeneity in Wnt signaling still exists within separate cells within a colon cancer [33,34]. Interestingly, colon cancer cells with high Wnt activity express stem cell markers and have higher clonogenic capacity and tumorgenicity, whereas cells with low Wnt activity express higher levels of differentiation markers and are unable to give rise to new tumors [22]. In this light, it is interesting to note that Lgr5 is a Wnt target gene [35], exclusively expressed on the normal intestinal stem cells [36] and could thus also be a CSC marker. Interestingly, human colon CSCs were found to express Lgr5 [22]. In addition, tumorigenesis in mice is much more effective in Lgr5^+^ stem cells as compared to more differentiated cells [21]. However, formal evidence that Lgr5 identifies colon CSCs is still lacking and awaits the generation of reliable antibodies that can be used to isolate Lgr5^+^ tumor cells.

## FUNCTION OF CSC MARKERS AND THEIR RELEVANCE TO CSC BIOLOGY

Most of the markers used for colon CSC isolation are chosen either because they are expressed in normal stem cells or were found to identify CSCs in other malignancies, either hematological or solid. The disadvantage of choosing markers in this fashion is that the functional effect of expression of the marker in CSCs is usually unknown. Also, the expression level (high versus low) used for identifying CSCs sometimes differs between tissue types [37]. Therefore, the choice of these markers is largely based on an “educated guess”.

For instance, breast cancer cells expressing either no or low levels of single chain sialoglycoprotein CD24 in combination with CD44-positivity have been identified as CSCs [3], whereas in pancreas and colon, the CD24^high^ cells were shown to contain the tumor-initiating fraction [10,17]. CD24, first described as a cell surface marker for several haematopoietic cell populations, is known to play a role in cell-cell interaction [38,39], adhesion and proliferation [40,41]. Many cancers were found to over-express CD24 [42-44], indicating that the protein can play an important role in tumorigenesis. However, its role in CSC biology remains undefined, especially because the expression level by which the CSC should be selected differs between tissue types. Together with CD24, CD29-positivity has been used to identify the colon CSC population [17]. Cell surface receptor CD29, like CD24, mediates cell to extracellular matrix adhesion and can promote cancer progression by inducing invasion, migration and metastasis [45,46]. Its function in CSC biology has not been studied yet.

The choice for CD166 as a marker for CSCs was based on the heterogeneous expression of CD166 in colon carcinoma in combination with the finding that its expression correlates with a poor clinical outcome in colorectal cancer (CRC) patients [47]. Also CD166 is involved in regulation the formation of cell-cell contacts [48,49]. Even though the function of the debated CSC marker CD133 remains unknown, its presence in plasma membrane protrusions [50,51] as well as its ability to interact with plasma membrane cholesterol [52] suggests that the protein might play a role in establishing and maintaining plasmamembrane protrusions. This suggests that CD133 could play a role in cell polarity and migration [53] via cell-cell and cell-matrix interactions. In contrast, knockdown of CD133 in colon cancer cell lines or primary colon cultures did not influence the proliferation, migration, invasion, clonogenic and tumorigenic formation capacity [19,54], indicating that the protein itself is not required for maintenance of the CSC state.

Interestingly, most of the CSC markers were chosen based on expression level instead of potential function in CSCs and most of them seem to play a role in inducing and/or maintaining cell-cell or cell-matrix contact. A worrying aspect of such CSC markers is that enhanced attachment could in part explain why cells expressing these markers are more capable of growing in a new environment. Xenograft growth is currently the accepted/compulsory method to prove cancer stemness, but identification of adherence factors as CSC markers could simply be a reflection of the need for adherence in the assay itself. For this reason, it would be favorable to identify CSCs by markers that have a clear function in CSC biology.

CSC marker CD44 is one of those markers that might have a more functional role in CSC biology. For instance, knockdown of CD44 in primary colon cancer cell lines reduces clonogenicity in vitro and tumorgenicity in vivo [19]. CD44 is known to be involved in many cellular processes like survival, growth, differentiation and survival. As an important adhesion molecule, CD44 plays a major role in cancer cell migration. Several different splice variants are known for CD44. Interestingly, the ectodomain of one of its variants, CD44v6, was found to be required for c-Met activation in several tumors [55,56]. Moreover, hepatocyte growth factor (HGF), the ligand for c-Met, can restore the CSC phenotype in more differentiated colon cancer cells [22] and seems to play a major role in maintaining a CSC state. Therefore, CD44 itself may have a role in regulating stemness in CSCs and thus may be a functional cell surface CSC marker.

A more functional read-out of stemness in cancer cells is the activity of the ALDH1 enzyme. This enzyme, which oxidizes intracellular aldehydes, identifies the cells that are more resistant to alkylating agents [57-59]. Therefore, cells that have high ALDH1 activity are better protected against oxidative insults. In concordance, CSCs are thought to be more resistant to therapeutic agents [60-67]. In addition, ALDH1 convert retinol to retinoic acid. The effect of retinoids on cells is very dependent on the differentiation stage in which there are, but in hematopoietic stem cells (HSCs), retinoids have been shown to enhance self-renewal [56,68]. Therefore, high ALDH1 activity can have a dual role in maintaining a CSC state.

Another functional marker for CSCs, as recently identified by our group, is Wnt activity. For normal intestinal stem cells, Wnt activity is essential for maintaining stemness and crypt survival [69,70]. Important players in the Wnt signaling cascade, the tumor suppressor adenomatous polypolis coli (APC) and ß-catenin, are frequently mutated in colon cancer. As a consequence, ß-catenin will not be degraded by the APC complex and accumulation of ß-catenin in the nucleus and consequent activation of Wnt target genes occurs [71]. However, despite the presence of these activating mutations, tumors display heterogeneous expression of nuclear ß-catenin [33,34], indicating that alternative regulation of Wnt signaling is taking place. Cells with high Wnt activity represent the CSCs within the tumor. Importantly, extrinsic cues, like HGF produced by surrounding myofibroblasts in the stroma, were shown to modulate the Wnt signaling activity as well, providing a connection between the stroma and the CSCs [22].

As described above, some functional markers have been identified, but more will be needed. In addition, the currently used cell surface markers should be more investigated to discover their role in CSC biology.

## WHY TARGET CSC?

Reliable markers that identify CSCs will pave the way to better understanding of signaling pathways and other regulatory mechanisms that determine stemness and differentiation of CSCs. As CSCs are considered to be the driving force behind tumor growth, therapies will have to focus on strategies that include targeting of CSCs. However, radio- or chemotherapy of cancer often incompletely eradicates tumor cells [60-67] and this is thought to be due to a selective survival advantage of CSCs, which could explain relapse of the tumor after many years. For example, studies have shown that colon CSCs are more resistant to treatment with 5-FU or oxaliplatin [16,72]. In addition, when CRC cell lines were treated with 5-FU or oxaliplatin in vitro, an increase in CD133^+^CD44^+^ cells was observed [73], indicating that the CSC fraction was enriched and thus resistant to these therapeutics. Recurrence of colon cancer and appearance of distant metastasis many years after initial treatment are therefore hypothesized to be caused by residual CSCs. So, by targeting the CSCs specifically, it should be possible to obtain more complete degeneration of the tumor. Obviously, combination therapies that target both CSCs and more differentiated progeny will in the end be more efficient for use in the clinic. Especially as new studies have shown that factors produced by the microenvironment can revert differentiated cells back to a more stem cell-like state [22], indicating that killing the CSCs alone might not be sufficient to diminish tumor growth.

From a more scientific perspective, killing CSCs selectively in growing tumors could validate the currently favored hypothesis that CSCs are the only clonogenic cells within tumors. As indicated above, the current xenotransplantation assays define the cells that are capable of initiating tumor growth in immunocompromised mice. However, it is currently unclear whether these cells are also required to maintain growth of an existing tumor. Therefore, ablating CSCs selectively in growing tumors in vivo will give more insight into their biological role in tumors as well as their flexibility/plasticity.

## TARGETING CSC MARKERS

A strategy to target CSCs is exploiting the presence of their cell surface markers. Membrane proteins can be targeted directly or used to internalize a death-inducing compound. For instance, in AML blasts, the first strategy was employed. One of the problems in AML is that blast cells are arrested in more progenitor-like state and do not fully differentiate. The CSC marker CD44 is known to play a role in normal myeloid differentiation, as this can be inhibited by CD44 blocking antibodies [74,75]. Treatment of AML blasts in vitro with CD44-activating monoclonal antibodies relieves the differentiation blockade. Moreover, in some cases, the CD44-activating antibodies could decrease proliferation and increase apoptosis of the blasts [76]. Additionally, leukemic stem cells (LSCs) could be killed in vivo when mice transplanted with AML cells were treated with the activating CD44 antibody H90. Ligation of CD44 inhibited the migration of LSCs to their niche, but also affected LSCs intrinsically, because repopulation capacity was severally affected afterwards [16,77]. Combined, this suggests that targeting CD44 could be a good strategy to attack CSCs.

The use of cell surface markers to guide toxic insults was employed by Wang et al. who targeted the CSC marker CD133 in glioblastoma (GBM). They conjugated an anti-CD133 antibody to single walled carbon nanotubes (SWNTs), which were developed to serve as an alternative option for localized hyperthermia treatment in comparison to gold-based nanomaterials [78,79]. Exposing CD133^+^ GBM cells to these anti-CD133-SWNTs induced internalization of the SWNTs whereas CD133^−^ cells did not take up the SWNTs. Exposing the cells to 808-nm near-infrared (NIR) laser light induced specific killing of the CD133^+^ cells. In vivo treatment of CD133^+^-SWNT-treated GBM cells by NIR laser induced photothermolysis also significantly reduced tumor growth [80].

## TARGETING DIFFERENTIATION AND SURVIVAL SIGNALING IN CSC

Since conventional therapies do not suffice in killing all tumor cells, the addition of a sensitizer targeting a pathway responsible for resistance of CSCs or inducing differentiation could overcome this problem. For instance, colon carcinomas are producing IL-4 that is responsible for upregulation anti-apoptotic molecules in these tumors. Inhibiting IL-4 by blocking antibodies sensitizes the cells for killing by 5-FU and oxaliplatin [16]. Also induction of differentiation in colon CSCs by exposing these cells to Bone Morphogenetic Protein 4 (BMP4), which can initiate a differentiation program as well as mediate apoptosis [81], sensitizes colon cancer cells to killing with 5-FU or oxilaplatin in vivo, resulting in complete and long term regression of colon xenografts [82].

Also other signaling pathways were found to be involved in maintaining CSCs and therefore are thought to be essential for tumor growth. For instance, Hedgehog(HH) signaling is suggested to play a role in CSCs of several tumor types, like glioblastoma [83] , breast cancer [84], pancreatic cancer [85], multiple myeloma [86] and colon cancer [87]. To activate HH signaling, HH binds to the receptor Patched leading to the release of Patched-mediated repression of Smoothened (SMO) and to activation of the downstream Gli transcription factor family. In CD133^+^ advanced/metastatic colon cancer cells, higher levels of Gli1, a measure for HH activity, were found, compared to CD133^−^ cells. Inhibition of HH signaling in primary colon cancer cell lines by knockdown of SMO reduces proliferation and induces apoptosis in vitro. In addition, in vivo tumor growth is abrogated by inhibition of HH signaling, because of reduced survival of CSCs [87].

Also Notch signaling has been identified as an essential pathway for the maintenance of the stem-cell like state of colon CSCs [88]. An important component of the Notch pathway is delta-like 4 ligand (DLL4). By inhibiting DLL4 with human monoclonal antibody 21M18 in colon carcinoma xenografts, the tumor growth as well as the CSC frequency, measured by the amount of ESA^+^CD44^+^CD166^+^ cells is decreased compared to control. Interestingly, even though treatment of the xenografts with irinotecan, a chemotherapeutic often used in colon cancer, slowed down tumor growth, the frequency of ESA^+^CD44^+^CD166^+^ cells and the clonogenicity was increased. Combination treatment of irinotecan with anti-hDLL4 reduced again the tumor growth and stem cell frequency, at even higher levels than the anti-DLL4 treatment alone [89]. This indicates that inhibiting Notch signaling reduces CSC frequencies and sensitizes tumor cells for irinotecan treatment.

## GENE SUICIDE THERAPY FOR TARGETING CSC AS PROOF OF PRINCIPLE

As mentioned above, many studies support the existence of CSCs. However, sorting techniques to isolate CSCs, as well as xenotransplantation assays needed to prove stemness, also disrupt the tumor microenvironment and likely introduce a selection bias. Therefore, it is difficult to prove that CSCs unequivocally exist. To study the actual role of CSCs in tumor growth in vivo, gene suicide therapy specifically against CSCs are needed. Using this strategy, suicide genes like the herpex simplex virys thymidine kinase (TK), diphtheria toxin or pro-apoptotic genes are placed under the regulation of a CSC-specific promoter. The activity of the suicide genes is then regulated either by a prodrug such as ganciclovir for TK or by combination with a genetic control mechanism, for instance using a stop cassette flanked by loxP sites so that suicide gene expression can be induced upon removal of the floxed cassette. CSCs can then be specifically targeted and the effect on tumor growth can be studied.

A method to target CSCs by a suicide gene that is independent of cell division is making use of Diphtheria Toxin A (DTA) gene. The DTA gene encodes for an adenosine diphosphate (ADP) ribosyltransferase which is responsible for ADP-ribosylation of elongation factor 2. The effect of this ribosylation is that protein synthesis is inhibited and cell death is induced [90]. The advantage of this system is that only one molecule of DTA per cell is sufficient to induce cell death [91]. A recent study was published that made use of expressing DTA under a CSC marker. In this study, mouse glioma-initiating cells (GICs) were generated by overexpressing SV40 Large T antigen and oncogenic Ras in neural mouse stem cells. To be able to specifically eradicate GICs, these cells were genetically modified to carry the DTA gene by a floxed-lacZ cassette under the control of the CD133 locus as well as a CreER construct under the expression of a ubiquitous promoter. Injection of these GICs resulted in tumor formation that matched human glioma phenotypically. In addition, the tumors displayed a subset of cells that were CD133^+^. When the GICs were ablated from CD133^+^ cells by addition of tamoxifen in vitro and subsequently injected in vivo, the tumor formation was not affected, showing that CD133^+^ cells are not required for tumorigenesis in this model [92]. Whether this is also the case for colon cancer growth remains to be established. However, these findings could significantly change the CSC dogma and suggest that CSC-specific therapies may not be sufficient to treat cancer.

## CONCLUDING REMARKS

Several markers have been used to identify colon CSCs, such as CD24, CD29, CD133, CD166, but the function of these proteins in CSC biology has not yet been clarified. Interestingly, these CSCs markers are involved in cellular adherence and since the golden standard in this field to identify CSCs by their capacity to recapitulate the original tumor when xenotransplanted into immunocompromised mice, the presence of adherence markers can be exploited by these cells to form new tumors. Therefore, the role of CSCs markers in maintaining a stem cell state should be investigated and more functionally important markers should be identified, like CD44, ALDH1 activity and Wnt signaling. Combination of previously known and new markers could lead to more directed therapy that specifically targets CSCs. At therapeutic and scientific level, specific targeting of CSCs could give more insight into how and whether these CSCs maintain tumor growth and in which way we can eradicate them. Studies that target CSCs within a growing tumor by suicide gene therapy could give not only more insight into their ability to maintain tumor growth, but also into their flexibility/plasticity within a tumor. This is especially relevant as an increasing number of studies indicate a role for the microenvironment/niche in the maintenance CSCs, which could even be dominant. Although this role is currently ill-defined it could provide novel clues for CSC biology as well as therapy.
